# Structural Controlling of Highly-Oriented Polycrystal 3C-SiC Bulks via Halide CVD

**DOI:** 10.3390/ma12030390

**Published:** 2019-01-27

**Authors:** Zhiying Hu, Dingheng Zheng, Rong Tu, Meijun Yang, Qizhong Li, Mingxu Han, Song Zhang, Lianmeng Zhang, Takashi Goto

**Affiliations:** 1 State Key Laboratory of Advanced Technology for Materials Synthesis and Processing, Wuhan University of Technology, Wuhan 430070, China; huzhiying312@foxmail.com (Z.H.); daixu20100912@163.com (D.Z.); turong@whut.edu.cn (R.T.); liyangmeijun@163.com (M.Y.); lmzhang@whut.edu.cn (L.Z.); goto@imr.tohoku.ac.jp (T.G.); 2 Hubei Key Laboratory Advanced Technology of Automobile Parts, Wuhan University of Technology, Wuhan 430070, China; qizhongli@whut.edu.cn; 3 R&D, Ibiden Co. Ltd., 1-1 Kitagata, Ibigawa-cho, Ibi-gun, Gifu 501-0695, Japan; han_mingxu@ibiden.com

**Keywords:** 3C-SiC, chemical vapor deposition (CVD), orientation, surficial morphology, wettability

## Abstract

Highly-oriented polycrystal 3C-SiC bulks were ultra-fast fabricated via halide chemical vapor deposition (CVD) using tetrachlorosilane (SiCl_4_) and methane (CH_4_) as precursors. The effects of deposition temperature (*T*_dep_) and total pressure (*P*_tot_) on the orientation and surficial morphology were investigated. The results showed that the growth orientation of 3C-SiC columnar grains was strongly influenced by *T*_dep_. With increasing *T*_dep_, the columnar grains transformed from <111>- to <110>-oriented. The arrangement of <111>-oriented columnar grains was controlled by *P*_tot_. Lotus-, turtle-, thorn-, and strawberry-like surface morphologies were naturally contributed by different arrangements of <111>-oriented grains, and the deposition mechanism was discussed. The wetting behaviors of CVD-SiC samples by molten aluminum were also examined at 1173 K in a high vacuum atmosphere.

## 1. Introduction

The cubic polytype of SiC (3C-SiC) can be used as a structural material, particularly in harsh environments, due to its high hardness and strength, and good thermal shock resistance and corrosion resistance [1,2,3]. Generally, SiC bulks are densified using method such as pressureless sintering, spark plasma sintering, and liquid-phase sintering with additives (Al_2_O_3_, AlN, B, C, and B_4_C) [4,5,6,7,8]. However, these additives negatively affect the high-temperature properties, which may cause defects. Therefore, highly pure and dense SiC is in demand for industrial applications. For example, graphite materials with polycrystal 3C-SiC coatings are always used as heater or furnace walls in semiconductor annealing/doping furnaces, due to the good thermal shock resistance and corrosion resistance of 3C-SiC coating. During the doping process, melting aluminum (as the doping agent for controlling charge carrier mobility of raw materials) droplets are easily splashed to adhere to the surface of 3C-SiC (3C-SiC heater/wall). After the doping process, the 3C-SiC coating with poor wettability is easy to clean, whereas Al droplets are harder to remove from coating with a smooth surface. Chemical vapor deposition (CVD) is a promising technique for generalizing the use of SiC; CVD has been intensively studied and applied in annealing furnaces [9,10]. Thick and highly pure SiC is needed to satisfy the basic requirements and function under harsh environments. CVD is able to produce highly pure SiC films, whereas the deposition rates of SiC need to be considerably increased for fabricating bulky polycrystal SiC [11,12]. Monolithic 3C-SiC wafer with Φ80 mm diameter was prepared by CVD on a graphite plate with the ultra-fast speed of 930 μm/h in our group’s previous works [13,14]. The properties, such as corrosive resistance and wettability, are always determined by microstructure and surficial morphology of the deposits [15,16,17,18]. To the best of our knowledge, reports are lacking about controlling the surficial morphology and understanding the growth mechanism of the deposited SiC with thickness in the mm-order. Therefore, in this current study, we attempted to control microstructure and surficial morphology of highly-oriented 3C-SiC bulks by studying deposition temperature (*T*_dep_) and total pressure (*P*_tot_) via halide CVD. The growth mechanism of the deposits has been investigated via the crystallography of the grown 3C-SiC grains.

## 2. Materials and Methods 

Graphite plates (20 × 20 × 5 mm) were used as substrates for the growth of SiC in a chamber of a vertical type hot-wall CVD. The details of the CVD apparatus can be found in our previous report [13]. The deposition temperature (*T*_dep_) was 1573 to 1823 K. *T*_dep_ was calibrated by measuring the temperature near the top and bottom heating units by two W/Re thermocouples. An infrared thermometer (IR-AH, Chino, Tokyo, Japan), which focused on the surface of specimens, was set to monitor *T*_dep_. The total pressure (*P*_tot_) was controlled in the range of 1 to 40 kPa. The reactive gas featured a mixture of tetrachlorosilane (SiCl_4_, Aladdin industrial Co., Ltd, Shanghai, China, 99.50%), methane (CH_4_, Wuhan Xiangyun Chemical Industry Co., Ltd., 99.99%) were diluted in an H_2_ flow of 700 sccm (standard cubic centimeter per minute at standard temperature and pressure). The liquid SiCl_4_ was kept in a tank and conveyed via continuous bubbling methods with H_2_ as a carrier gas. The flow rates were tuned using a mass flow controller (MFC; D07-7, Sevenstar, Beijing, China). Before deposition, the graphite substrate was pretreated in dilution H_2_ flow of 2000 sccm for 5 min. The deposition time was 2 h. A cold trap surrounded by liquid nitrogen, a filter filled with activated carbon, and a NaOH spray scrubber were established to treat the exhaust gases. 

X-ray diffraction (XRD; θ-2θ) with Cu-*K*_α_ radiation (Ultima III, Rigaku, Tokyo, Japan) at 40 kV and 40 mA was carried out to examine the crystal phase as well as the preferred orientation. A field emission scanning electron microscope (FESEM, Quanta-FEG250, FEI, Houston, TX, USA, at 20 kV) was used to obtain the surface and cross-sectional microstructure of the specimens. A carbon/sulfur analyzer (CS-2000, Eltra, CITY, Germany) was applied to determine the carbon content. The silicon content was obtained by the chemical analysis of potassium fluorosilicate (K_2_SiF_6_) acid-base titration. The microstructure was observed by transmission electron microscopy (TEM; JEOL JEM-2100, Tokyo, Japan; 200 kV). The SiC samples were immersed into ethanol and ultrasonically cleaned for 10 min before the molten aluminum wetting experiment. Table 1 provides an overview of the deposition conditions and chemical composition (Si/C) of each sample.

## 3. Results

**Figure 1:**
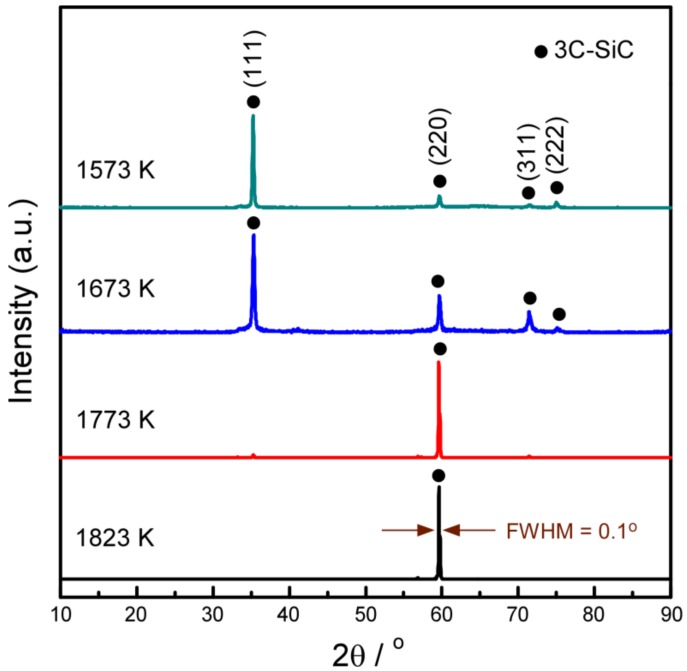
X-ray diffraction (XRD) patterns of the 3C-SiC deposit at various *T*_dep_.

Figure 1 shows the XRD patterns of the SiC deposited at *P*_tot_ = 4 kPa in the *T*_dep_ range of 1573 to 1823 K. SiC phase in all depositions is β-type (3C), corresponding to the results of chemical composition analysis. At *T*_dep_ = 1573 K, the relatively stronger 111 peak indicates the <111> orientation of the deposit. At *T*_dep_ = 1673 K, the intensity of 220 peak increased. At *T*_dep_ = 1773 K, the SiC film exhibited 220 peak indicating a significant <110> orientation. At *T*_dep_ = 1823 K, the full width at half maximum (FWHM) value of 220 diffraction was 0.1 degree.

The patterns show that the preferred orientation of 3C-SiC deposits was strongly influenced by *T*_dep_. With increasing *T*_dep_, the intensity of <110> diffraction peaks increased, whereas the intensity of the <111> peaks decreased. The chemical formation energy of the <110> orientation plane is higher than <111> orientation plane [19]^.^ Thus, the growth of {110} planes with relatively higher surface energy were inhibited at lower temperatures. The growth of {111} planes with relatively lower surface energy were promoted. The (111) crystals grew faster to cover (110) crystals. Conversely, it is more conducive to the growth (110) planes at higher temperatures. Changes in the preferred orientation of 3C-SiC can be understood in Van der Drift’s selective evolution model [20]. The model explains how random nucleation leads to the preferred orientation. During the primary stage of the nucleation and growth of crystal, randomly preferential growth orientations are observed. As the crystal growth proceeds, oriented crystals with the fastest growth rate possess the lowest chemical formation energy. These crystals cover other oriented crystal planes to form the final preferred orientation of deposit [20,21].

**Figure 2:**
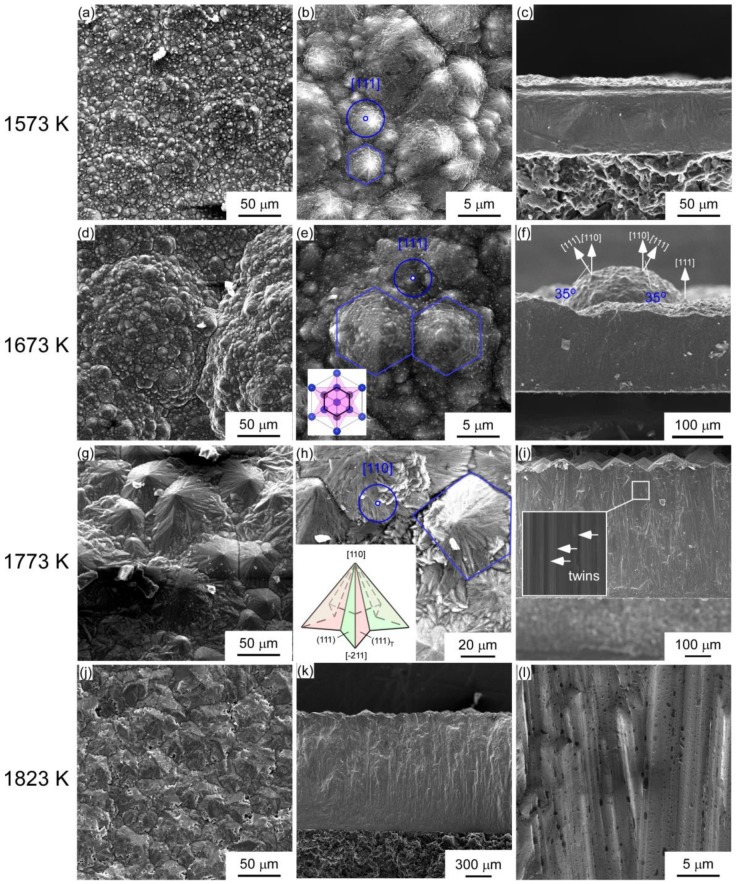
Surface and cross-section morphology of 3C-SiC deposits prepared at *P*_tot_ = 4 kPa, *T*_dep_ = (**a**)–(**c**) 1573 K, (**d**)–(**f**) 1673 K, (**g**)–(**i**) 1773 K, and (**j**),(**k**) 1823 K.

Figure 2 demonstrates the surface (Figure 2a,b) and cross-section (Figure 2c) morphology of SiC prepared at *T*_dep_ = 1573 K and *P*_tot_ = 4 kPa. Pyramid-like SiC grains with a hexagonal structure were obtained, which was ascribed to the six-fold symmetrical {111} planes of 3C-SiC. At *T*_dep_ = 1673 K (Figure 2d–f), the pyramid-like grains became larger. The change in the microstructure of the film at different temperatures depends on the orientation of the film. Pyramid-like grains with six-fold symmetry is the typical structure of <111>-oriented face-centered cubic crystal, producing the effect of the antiphase domain or twins, as shown as the inset in Figure 2e [22]. Figure 2 depicts the surface (Figure 2g,h) and cross-section (Figure 2i) morphology of 3C-SiC prepared at *T*_dep_ = 1773 K. The deposit is composed of many roof-like grains with five-fold symmetry. We found 100–200 μm-sized columnar grains perpendicular to the substrate in the cross-section of the film (Figure 2i) and twins were found in the inset. The {110} planes of SiC columnar grains were parallel to the substrate, growing continuously and forming a roof-like morphology. In recent research, we found that the {110} planes of SiC are caused by the twin-plane reentrant edge mechanism [14]^.^ The inset in Figure 2h illustrates a schematic of the five-fold symmetry roof-like grains, formed by stacking twinned surfaces. With further increases in *T*_dep_ to 1823 K, the <110>-oriented film consisted of roof-like grains sized ~20 μm with about a nanoscale gap width at the grain boundaries (Figure 2j–l). A number of voids sized ~0.5 μm were found between the grain boundaries due to the excessive growth rate at high temperature [14], which was not observed for other temperatures. The SEM observation corresponds to the XRD results (Figure 1), indicating the deposition temperature strongly affected the orientation of the deposits. Komiyama developed a model for predicting the preferred orientation in CVD processes by assuming Langmuir-type adsorption, including adsorption and reaction reactants [23,24,25]. The model suggests that when the maximum rate of adsorption exceeds that of surface reaction, the growth rate is limited by surface reaction. In our case, though {110} planes have higher formation energy, higher deposition temperature had enough energy supply to form the corresponding planes. The decomposition rate of {111} planes exceeded the formation rate due to the excessive energy at higher deposition temperatures.

**Figure 3:**
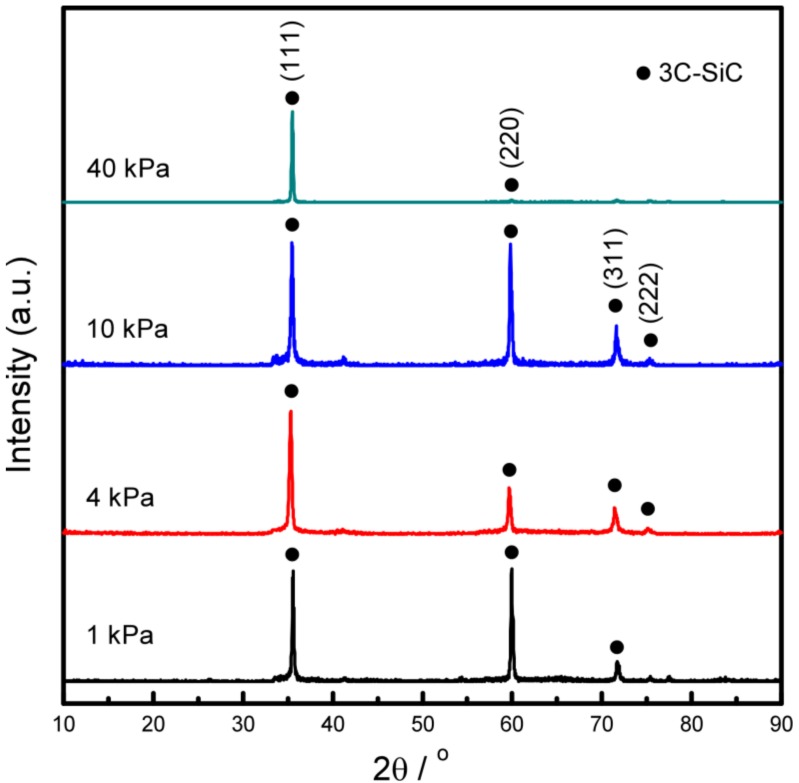
XRD patterns of the 3C-SiC deposits prepared at *T*_dep_ = 1673 K at various *P*_tot_.

The XRD diffraction patterns of the SiC specimen deposited with various *P*_tot_ are shown in Figure 3. The presence of all sharp diffraction peaks suggests that the deposits were well-crystallized. With decreasing *P*_tot_ from 40 kPa to 1 kPa, <111> orientation transformed to <111>-<110> co-orientation.

**Figure 4:**
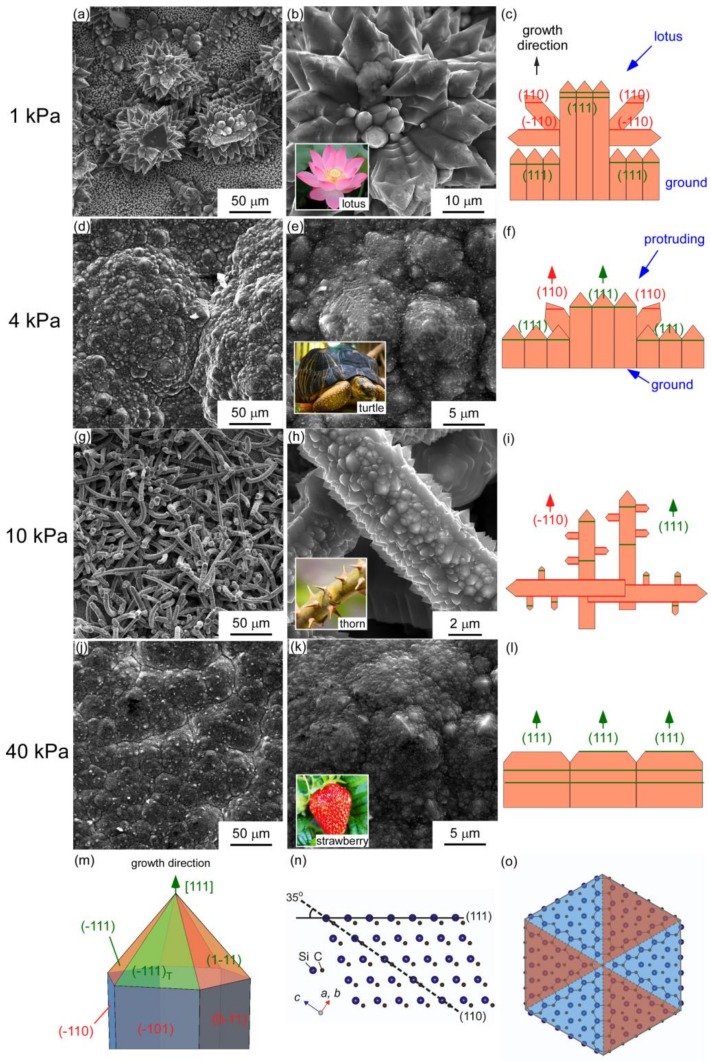
Surface and cross-section morphology of 3C-SiC films prepared at *T*_dep_ of 1673 K, *P*_tot_ of (**a**)–(**c**) 1 kPa, (**d**)–(**f**) 4 kPa, (**g**)–(**i**) 10 kPa, and (**j**)–(**l**) 40 kPa, and (**m**) morphology of single crystal. (**n**) Schematic diagram of the relationship between the (110) and (111) planes in 3C-SiC lattice; (**o**) the hexagon growth of 3C-SiC (111) films.

“Lotus in the ground” was exhibited in the surficial morphology at *T*_dep_ = 1673 K and *P*_tot_ = 1 kPa (Figure 4a,b). Both the lotus and the ground consisted of <111>-oriented columnar grain with a six-fold symmetry sharp tip, which is common in CVD 3C-SiC [26,27]. The nature of the <111>-oriented grain is shown in Figure 4m,o. Figure 4c displays the arrangement of the grains: the leading <111>-<110> co-orientation of the deposit. The angle between (111) and (110) planes is depicted in Figure 4n. Protruding in the ground were pyramids with six-fold symmetry, resembling a turtle shell, observed at *P*_tot_ = 4 kPa in Figure 4d,e. Figure 4f illustrates the structural details of the turtle shell. The hexagonal morphology of 3C-SiC (111) grains could be explained by TB analysis as shown in Figure 4o. At *P*_tot_ = 10 kPa, the deposit shows a thorn-like surficial morphology (Figure 4g). The schematic diagram of the arrangement is shown in Figure 4i. Thorn-like whiskers are the <111>-oriented columnar crystal with small <111>-oriented grains growing on the sides of “stem”. The whiskers were randomly arranged in the vertical and horizontal directions, so that the deposit also exhibited <111>-<110> co-orientation as shown as Figure 4i. At *P*_tot_ = 40 kPa, (Figure 4j,k), the relatively smoother surface of deposit shows a strawberry surface morphology that consisted of hexagons, which is the typical structure of <111>-orientation. Figure 4l is a schematic configuration diagram of the film growth at *P*_tot_ = 40 kPa. These SEM observations also correspond to the XRD results (Figure 3), indicating the morphology of the deposits strongly depended on *P*_tot_. Generally, the amount of nucleus in the CVD growth process is controlled by the concentration of the precursors. In this work, increasing *P*_tot_ led lower reactant concentrations. At *P*_tot_ = 1 kPa, the high concentration of the reactant created multiple nucleus adsorption sites, leading to the dense structure of the deposit. With decreasing concentration at *P*_tot_ = 4 kPa, there was not enough reactant pieces to grow a large lotus, which “degenerated” to protruding. A thorn-like whisker structure formed at low concentrations with further decreasing of *P*_tot_ at 10 kPa [28]. Such whisker-like structures preferentially nucleate on crystal planes with high concentration of defects and then nucleate and grow in random directions. At *P*_tot_ = 40 kPa, the three-dimensional (3D) growth model transformed to the two-dimensional (2D) model caused by extremely low reactant concentration. The grains are preferentially grown in the surface direction that formed a dense structure again.

**Figure 5:**
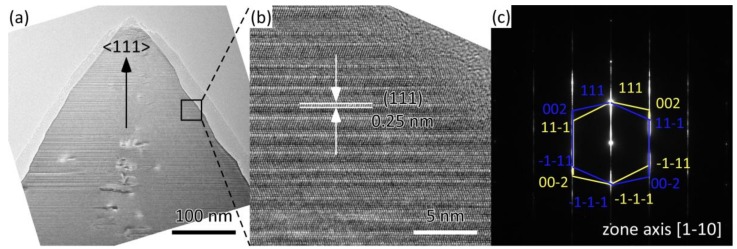
Transmission electron microscopy (TEM) images of sample B1 prepared at *P*_tot_ = 4 kPa, and *T*_dep_ = 1673 K. (**a**) the cross-sectional TEM BF images; (**b**) the magnified area of the BF image of the tip; (**c**) the SAED image for sample B1.

Figure 5 shows results of the transmission electron microscopy (TEM) analysis of the tip of an <111>-oriented columnar crystal in 3C-SiC sample B1 prepared at *T*_dep_ = 1673 K and *P*_tot_ = 1 kPa. Figure 5a shows the cross-sectional TEM bright-field (BF) images. A magnified area of the BF image of the tip is shown in Figure 5b. The lattice distance along the tip direction is approximately 0.25 nm, which is in agreement with the *d*-value of the 3C-SiC (111) plane. The Selected Area Electron Diffraction (SAED) (Figure 5c) was indexed as two sets (yellow and blue) of well-defined (111) twins, which are along the zone axes of [1–10] and [−110], respectively. The elongated spots imply a large amount of twins and stacking faults in the <111> direction.

**Figure 6:**
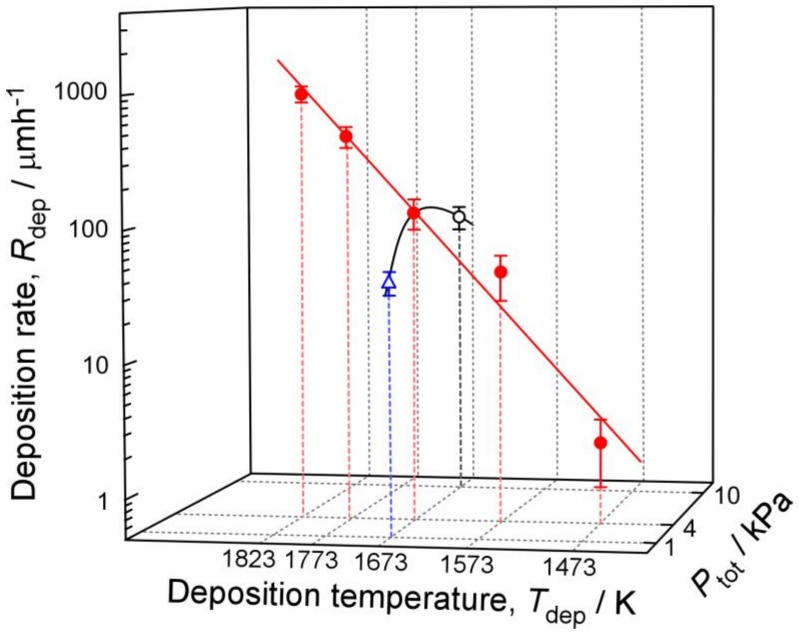
Effects of *T*_dep_ and *P*_tot_ on *R*_dep_.

Figure 6 shows the dependence of the deposition rate on *T*_dep_ and *P*_tot_. *R*_dep_ exponentially increases with increasing *T*_dep_. Generally, the *R*_dep_ increases with the gas concentration of precursors; higher *R*_dep_ may also increase with *P*_tot_. However, in this study, the maximum *R*_dep_ was obtained at *P*_tot_ = 4 kPa. For above than 4 kPa, the *R*_dep_ decreased due to the homogeneous reaction in the gas phase. In this study, the deposition rate changed with *P*_tot_ and showed a maximum *R*_dep_ of 731 μm^−1^ at *P*_tot_ = 4 kPa.

**Figure 7:**
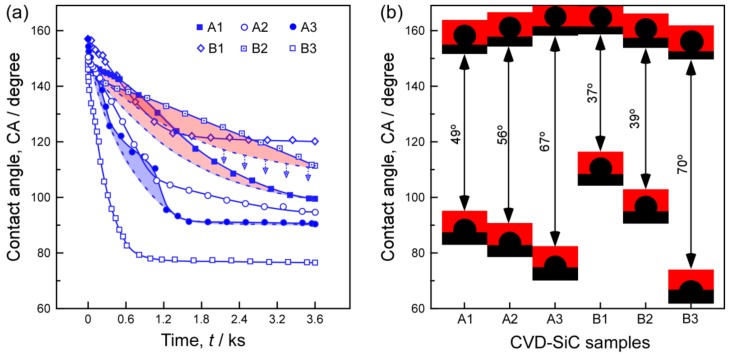
Wettability of the CVD-SiC samples by molten aluminum with time. (**a**) contact angles changes with time; (**b**) the variations in initial (upper panel) and final (bottom panel) contact angles.

The wetting behaviors of the CVD-SiC samples were examined at 1173 K under a high vacuum (~3 × 10^−4^ Pa) atmosphere using the improved sessile drop method, where liquid aluminum (Al 99.99 wt %, typically with mass of 0.1 g) sessile drop was formed by an alumina tube and then dropped on the surface of SiC substrates. The testing furnace consisted of a tantalum cylindrical heating element with a Mo isolating shield, a thermocouple near the alumina tube for temperature monitoring, an evacuating system with a rotary pump, a turbo molecular pump for creating a strong vacuum, a He-Ne laser for illumination, and a high resolution charge coupled device (CCD) camera to collect high-definition drop profiles. The moment at which the liquid Al contacted the surface of SiC and formed a droplet was defined as the start of the wetting process (i.e., *t* = 0 s in the wetting curve). Subsequent photographs were captured at certain time intervals the total photographing time was 3.6 ks. A detailed description of the apparatus installation and procedure was previously published [29]. Finally, the contact angle (CA) was calculated using an axisymmetric drop shape analysis program based on the captured drop profiles. Figure 7a shows the wetting behavior of pure Al on the oriented SiC films with respect to wetting duration at a wetting temperature of 1173 K, and Figure 7b depicts the variations in initial (upper panel) and final (bottom panel) contact angles. The shaded area in Figure 7a indicates that the wetting behaviors are probably affected by the SiC anisotropic surface that hinders the wetting process (especially for samples A1, A2, and B2). As shown as Figure 7b, all samples exhibited larger initial contacted angles up to 160 degree. We must notice that sample B1 and B2 with coarse surface also exhibited final contact angles larger than 100 degree, whereas the final contact angles (3.6 ks) of SiC with smooth surface were about only 60 degree in previous reports [30,31]. Sample B1, with a lotus-like surface morphology, had the largest contact angle both at the initial and final times. Sample B1 should be adequate for application in high temperature furnaces in the semiconductor industry.

## 4. Conclusions

Highly-oriented 3C-SiC bulks were fabricated using tetrachlorosilane (SiCl_4_) and methane (CH_4_) as a precursor via halide CVD. The orientation and microstructure are strongly dependent on the deposition temperature (*T*_dep_) and total pressure (*P*_tot_), respectively. With increasing *T*_dep_, the columnar grains and the dense deposits transform from <111>- to <110>-orientation. Lotus-, turtle-, thorn-, and strawberry-like surficial morphologies formed as a result of different arrangements of <111>-oriented grains, which are controlled by the concentration of the reactant. Sample B1, with lotus-like surficial morphology, had the largest contact angle with molten aluminum at 1173 K, both at the initial and final times. The larger contact angle caused a poor adhesive force of Al droplets with 3C-SiC coating, leading Al droplets to be easy to remove from the SiC coating.

## Figures and Tables

**Table 1:** Deposition conditions and chemical composition (Si/C).

Sample	A1	A2	A3	A4	B1	B2	B3
*T*_dep_ (K)	1573	1673	1773	1823	1673	1673	1673
*P*_tot_ (kPa)	4	4	4	4	1	10	40
Flow rate of H_2_	700 sccm						
Deposition time	2 h						
Si/C	1.02	0.97	1.01	1.00	0.98	1.02	1.01
Thickness (μm)	85	201	492	1462	80	195	184
